# A Manual of Practical Therapeutics

**Published:** 1886-11

**Authors:** 


A MANUAL OF PRACTICAL THERAPEUTICS, considered with reference to articles of the Materia Medica, by Edward John Waring, C. I. E., M. D., Fellow of the Royal College of Physicians, London; Surgeon Major (retired) in Her Majesty's India army. Edited by Dudley W. Buxton, M. D., B. S., London, member of the Royal College of Physicians; assistant to the Professor of Medicine at University College, London, and Administrator of Anesthetics at University College Hospital and the Dental Hospital of London. Fourth edition. Philadelphia: P. Blakiston, Son & Co., No. 1012 Walnut street. 1886.

After bringing the information in each edition up to date, and curtailing the superfluous matter, the author confidently expects that the practical usefulness of this work will be materially increased. A feature in connection with the index of diseases, by which the reader learns what articles of the materia medica are used in the different affections, is of very doubtful propriety. If a proper judgment is formed by a physician as to the nature of the disease, he ought not to require such a guide for determining upon the remedy, and a mistake in diagnosis, or the use of this list by the unprofessional, may prove disastrous. The concise and practical character of the therapeutic suggestions commend the book to the favorable consideration of medical men.

